# Analysis of Salivary Neuropeptides in Anxiety and Depression Using the Luminex MAGPIX® System

**DOI:** 10.7759/cureus.67984

**Published:** 2024-08-28

**Authors:** Ida Kupcova, Lubos Danisovic, Sona Bernatova, Stefan Harsanyi

**Affiliations:** 1 Institute of Medical Biology, Genetics, and Clinical Genetics, Faculty of Medicine, Comenius University Bratislava, Bratislava, SVK; 2 Department of Psychiatry, Psychiatric Clinic, The University Hospital Brno, Brno, CZE

**Keywords:** salivary neuropeptides, depression, substance p, neurotensin, beta-endorphin, alpha-msh, oxytocin

## Abstract

Background: Anxiety and depressive disorders are highly prevalent mental health conditions, affecting millions worldwide. Advancements in neurobiology have identified the effects of various neuropeptides in modulating mood and stress responses. Some of the well-researched neuropeptides in plasma are oxytocin (OXT), alpha-melanocyte-stimulating hormone (alpha-MSH), beta-endorphin, neurotensin, and substance P. In this study, we used methods of liquid biopsy to acquire saliva samples to analyze the concentrations of neuropeptides associated with depression.

Methods: The study was conducted in Bratislava, Slovakia, from January to June 2022. Participants were 20 subjects treated for depression and anxiety without medication; the control group consisted of 20 healthy individuals with no personal history of depression or anxiety. Salivary samples were collected using buccal swabs to measure the concentrations of the examined neuropeptides. Laboratory analysis was based on detecting fluorescent signals performed on the Luminex MAGPIX® System (Luminex Corporation, Austin, Texas). Means and standard deviations were calculated for individual neuropeptide levels. To determine if there are statistically significant differences in neuropeptide levels between individuals with and without depression, independent t-tests and a one-way ANOVA were conducted.

Results: Our findings indicate a significant decrease in all studied neuropeptides in subjects compared to healthy controls. Reductions in mean levels were observed for OXT (7.3), alpha-MSH (3.9), beta-endorphin (2.9), neurotensin (15.1), and a 6.9-fold decrease for substance P. Alpha-MSH and beta-endorphin showed higher variability in measured levels within both groups.

Conclusion: The results of this study indicate that the levels of the studied salivary neuropeptides, OXT, alpha-MSH, beta-endorphin, neurotensin, and substance P, are statistically significantly reduced in individuals with depression compared to healthy controls.

## Introduction

Depressive disorders are among the most prevalent mental health conditions worldwide, affecting millions of people and posing significant public health challenges. According to the World Health Organization, over 280 million people suffer from depression globally [1,2]. Over the years, the prevalence has grown significantly, and the COVID-19 pandemic has impacted it. All groups of people, whether based on age, race, education, or socioeconomic status, suffer from depression; however, it is reported that low- and middle-income countries have worse capacities for diagnosis and treatment [3,4]. Advancements in neurobiology have shed light on the effects of various neuropeptides in modulating mood and stress responses [5]. Some of the well-researched neuropeptides in plasma are oxytocin (OXT), alpha-melanocyte-stimulating hormone (alpha-MSH), beta-endorphin, neurotensin, and substance P. Each of these molecules has been implicated in the regulation of anxiety and depressive disorders [6]. However, research using plasmatic concentrations requires drawing blood, which is invasive, while saliva samples are non-invasive and very easy to obtain. Liquid biopsy methods are becoming preferred in research, especially for their wide applicability. They could be used in biomarker examinations to enhance the management of many diseases, not only depression.

As the "love hormone," OXT is widely recognized for its role in social bonding and stress reduction. Elevated OXT levels have been linked to reduced anxiety and depressive symptoms, highlighting its potential as a therapeutic target for mood disorders [7]. Studies suggest that OXT's anxiolytic and antidepressant effects are mediated through its action on the hypothalamic-pituitary-adrenal (HPA) axis and its ability to enhance social interactions and emotional regulation [8,9]. A recent study on the association between hippocampus and amygdala volume and salivary OXT showed contradictory results [10].

Alpha-MSH, a peptide involved in the regulation of skin pigmentation, also plays significant roles in anti-inflammatory responses and energy balance [11]. Recent research indicates that alpha-MSH possesses neuroprotective properties and may mitigate neuroinflammation, which is often associated with depressive and anxiety disorders [12,13]. Its ability to modulate inflammatory pathways suggests potential benefits in treating neuroinflammatory components of these mental health conditions; however, this effect has not been proven yet.

Beta-endorphin, an endogenous opioid peptide, is crucial for pain modulation and stress management. It exerts natural analgesic effects and enhances a sense of well-being [14]. Elevated beta-endorphin levels are associated with improved mood and reduced symptoms of depression and anxiety [15]. Its role in the endogenous opioid system underscores its importance in developing new treatments for mood disorders, particularly those involving chronic stress and pain.

Neurotensin, a peptide that influences neurotransmission and dopamine pathways, plays a significant role in mood regulation, pain perception, and cognitive function [16]. Neurotensin's neuroprotective and psychotropic effects have been studied in the context of psychiatric disorders such as schizophrenia and depression [17,18]. Its ability to modulate dopaminergic activity positions it as a potential therapeutic agent for mood disorders.

Substance P is a neuropeptide involved in pain transmission and inflammatory responses. It is often elevated in chronic pain and inflammatory conditions, which are frequently comorbid with anxiety and depression [19]. Substance P's role in enhancing pain perception and stress responses makes it a critical target for developing treatments aimed at reducing both pain and mood disorder symptoms.

In this study, we used liquid biopsy methods to acquire saliva samples and analyze concentrations of neuropeptides associated with depression. We hypothesize that comparing salivary OXT, alpha-MSH, beta-endorphin, neurotensin, and substance P in patients with depression and controls could be as effective as comparing their serum levels.

## Materials and methods

Study design

This study was cross-sectional and included 20 participants with clinical depression (11 females, nine males) and 20 healthy control participants (12 females, eight males) without a personal history of depression or anxiety. All participants voluntarily enrolled in the study, which was conducted between January 2022 and June 2022 in Bratislava, Slovakia. The study aimed to compare neuropeptide levels between the two groups. Ethical approval for the study was granted by the local ethics committee (reference number: ULBGaKG-03/2022). All samples were collected following the Helsinki Declaration after submitting informed consent.

Study criteria

The inclusion criteria for the clinical depression group required participants to have a self-reported history of depression and to be scheduled for therapeutic interventions in psychiatric practice. For the control group, participants had to have no self-reported history of depression or anxiety disorders. The exclusion criteria for both groups included the active use of psychiatric medication and the presence of other psychiatric disorders. No subjects were excluded based on these criteria. The study's sample size was determined by participant availability and the time constraints of the study.

Study procedure and assessment

Saliva samples were collected in a time frame from 08:00 to 12:00 in sterile 2 mL polypropylene tubes (TPP, Trasadingen, Switzerland). After rinsing the mouth with water, the subjects used passive drooling to accumulate saliva in a collection tube. Subsequently, the samples were promptly frozen at −80 °C until evaluation. The saliva samples were thawed at 4 °C. The protease inhibitor cocktail set (Merck Life Science, Darmstadt, Germany) was utilized for salivary sample preparation in the first step. In this instance, sterile distilled water was employed as the working buffer for reconstituting the protease inhibitor cocktail set. Following reconstitution, 1 µL of the inhibitor was added to a 500 µL saliva sample. The mixture was then centrifuged at 10,000 rpm for 10 minutes, and the resulting supernatant was diluted 1:2 with assay buffer (Merck Life Science, Darmstadt, Germany).

To measure the concentration of selected neuropeptides in saliva samples, the MILLIPLEX MAP Human Neuropeptide Magnetic Bead Panel - Neuroscience Multiplex Assay (Merck Life Science, Darmstadt, Germany) was applied. Fluorescent signal detection was performed on the Luminex MAGPIX® instrument (Luminex, Austin, Texas) according to the manufacturer's instructions. The data were processed using Belysa™ software v1.2 (Belysa™ Immunoassay Curve Fitting Software, MilliporeSigma, Merck KgAA, Rahway, New Jersey).

Statistical analysis

The analysis involved calculating the means and standard deviations of individual neuropeptide levels. The Shapiro-Wilk test was applied to assess data normality, and Levene's test was used to verify the homogeneity of variances. Independent t-tests were conducted to evaluate statistically significant differences in neuropeptide levels between the depression and control groups, assuming normal distribution, independence of observations, and equal variances. A one-way ANOVA was also performed to compare group means under similar assumptions. Statistical significance was defined as a p-value ≤ 0.05. All statistical analyses were conducted using IBM SPSS Statistics for Windows, Version 29 (Released 2023; IBM Corp., Armonk, New York).

## Results

A total of 20 subjects with depression and 20 healthy controls were analyzed for salivary neuropeptide levels in the study. Table 1 presents the description of the included groups. All study participants were included. Stratification by age or gender did not prove statistically significant. The Shapiro-Wilk test was used to determine if the data followed a normal distribution. In the depression group, the test indicated a significant deviation from normality (p < 0.001), meaning the measured neuropeptide levels did not follow a normal distribution. In contrast, the control group showed p-values ranging from 0.30 to 0.395, suggesting that their data closely followed a normal distribution.

**Table 1:** Descriptive statistics of the study groups.

Group	Sex	n	Median Age (Years)	History of Depression
Subjects	Female	11	36	Yes
	Male	9	35.5	
Controls	Female	12	36	No
	Male	8	30	

**Figure 1:**
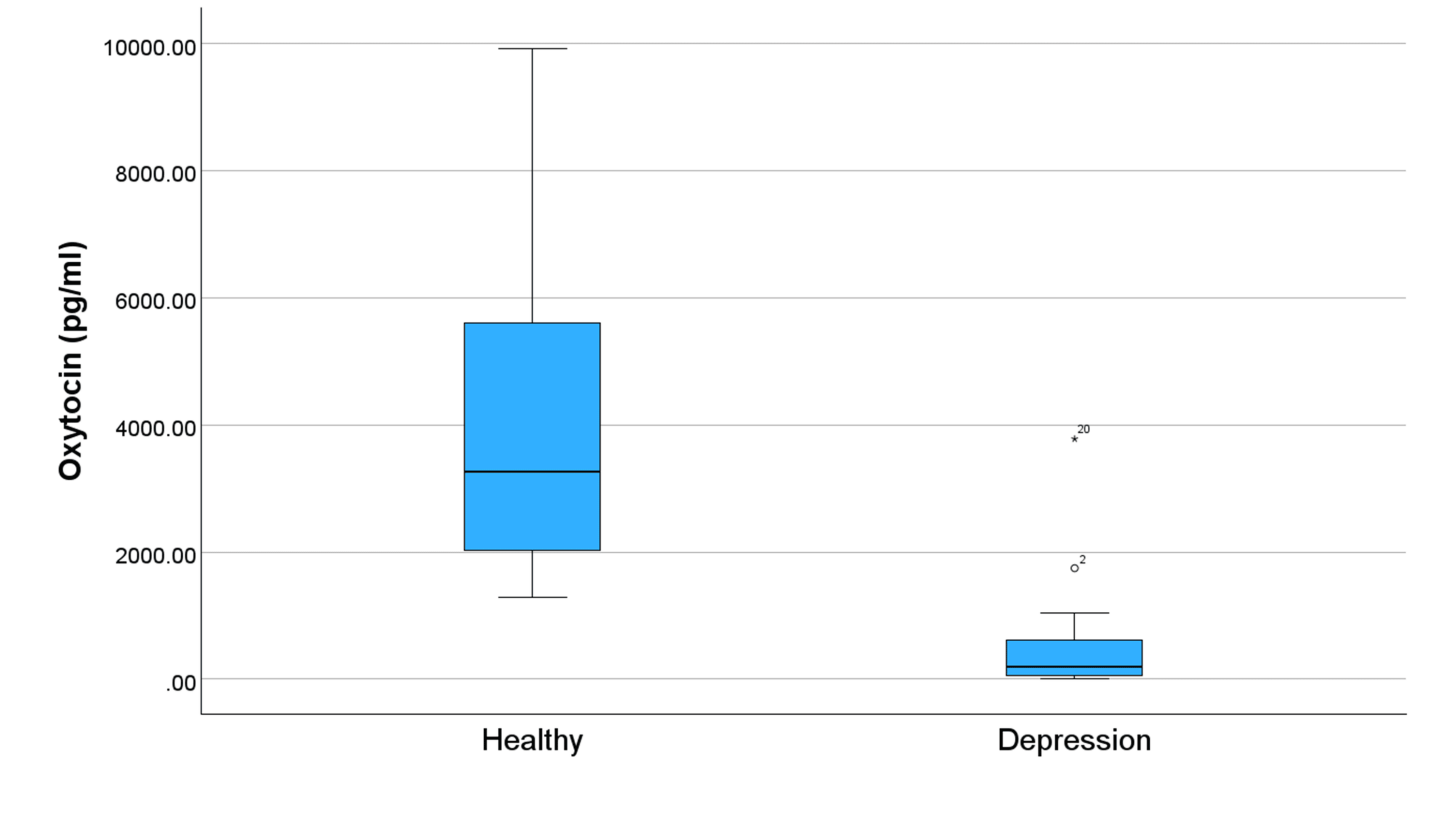
Distribution of oxytocin levels in individuals with depression and healthy controls. The symbols ⚬ and * represent outliers.

**Figure 2:**
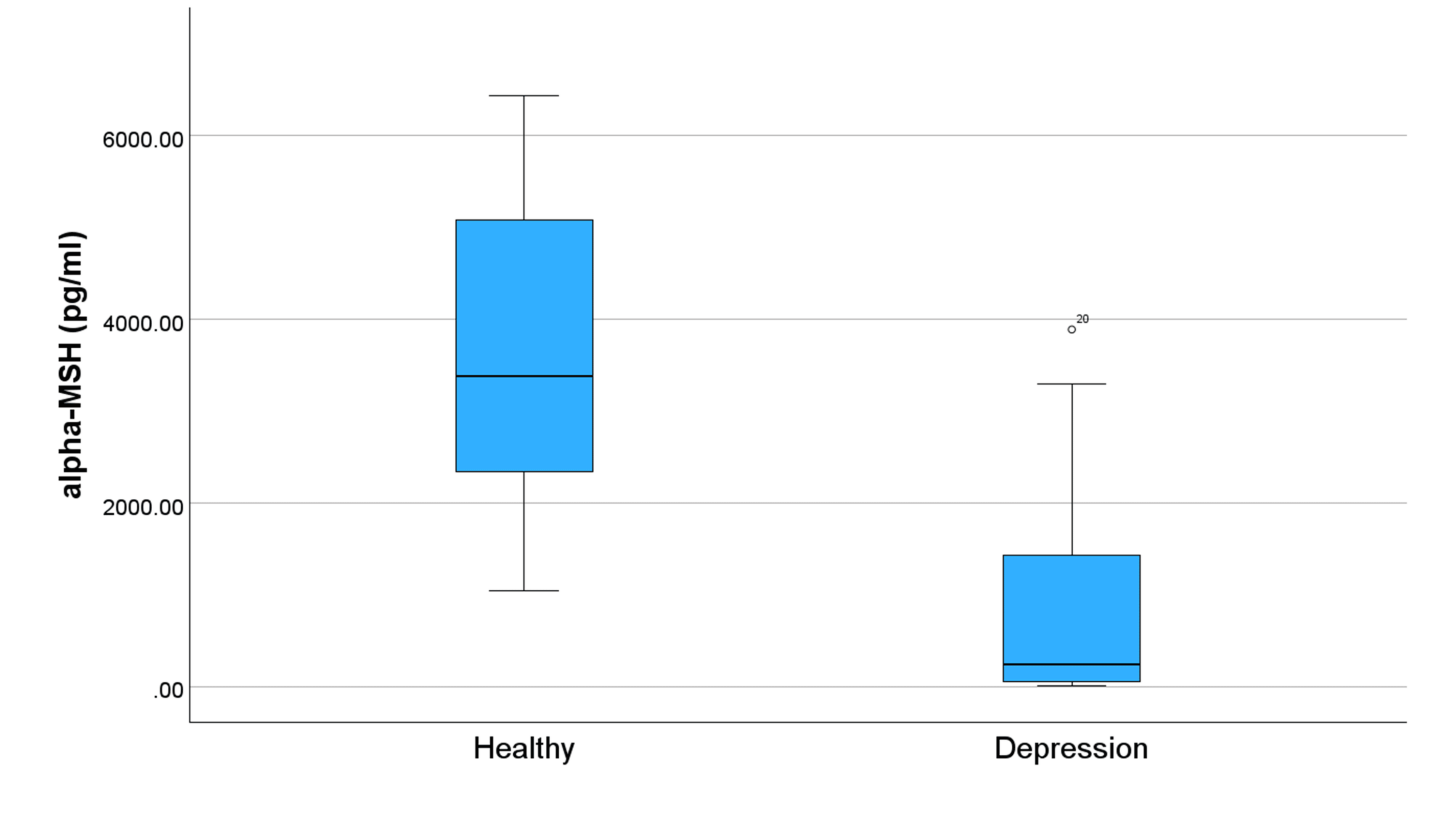
Distribution of alpha-MSH levels in individuals with depression and healthy controls. The symbol ⚬ represents an outlier.

**Figure 3:**
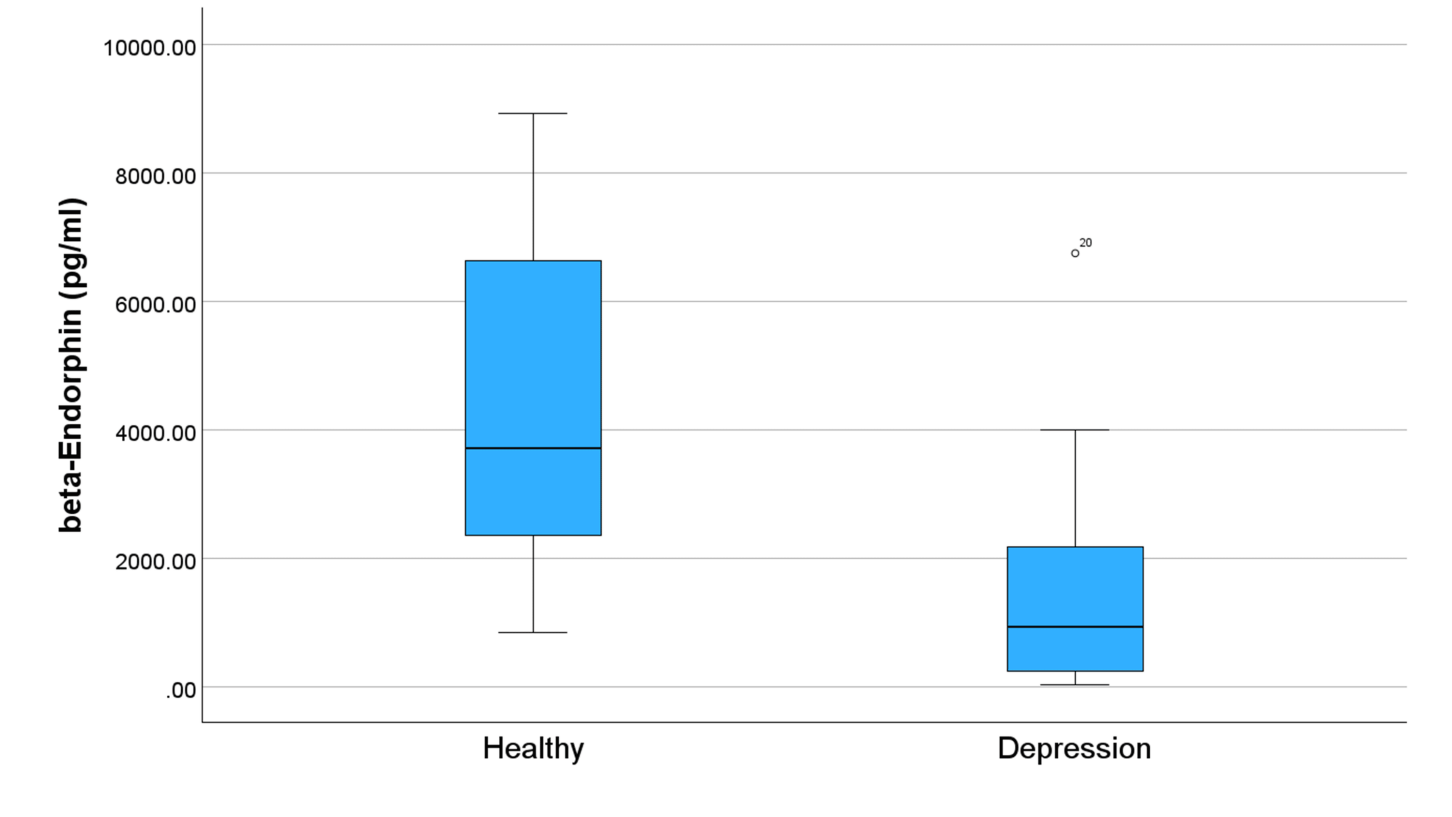
Distribution of beta-endorphin levels in individuals with depression and healthy controls. The symbol ⚬ represents an outlier.

**Figure 4:**
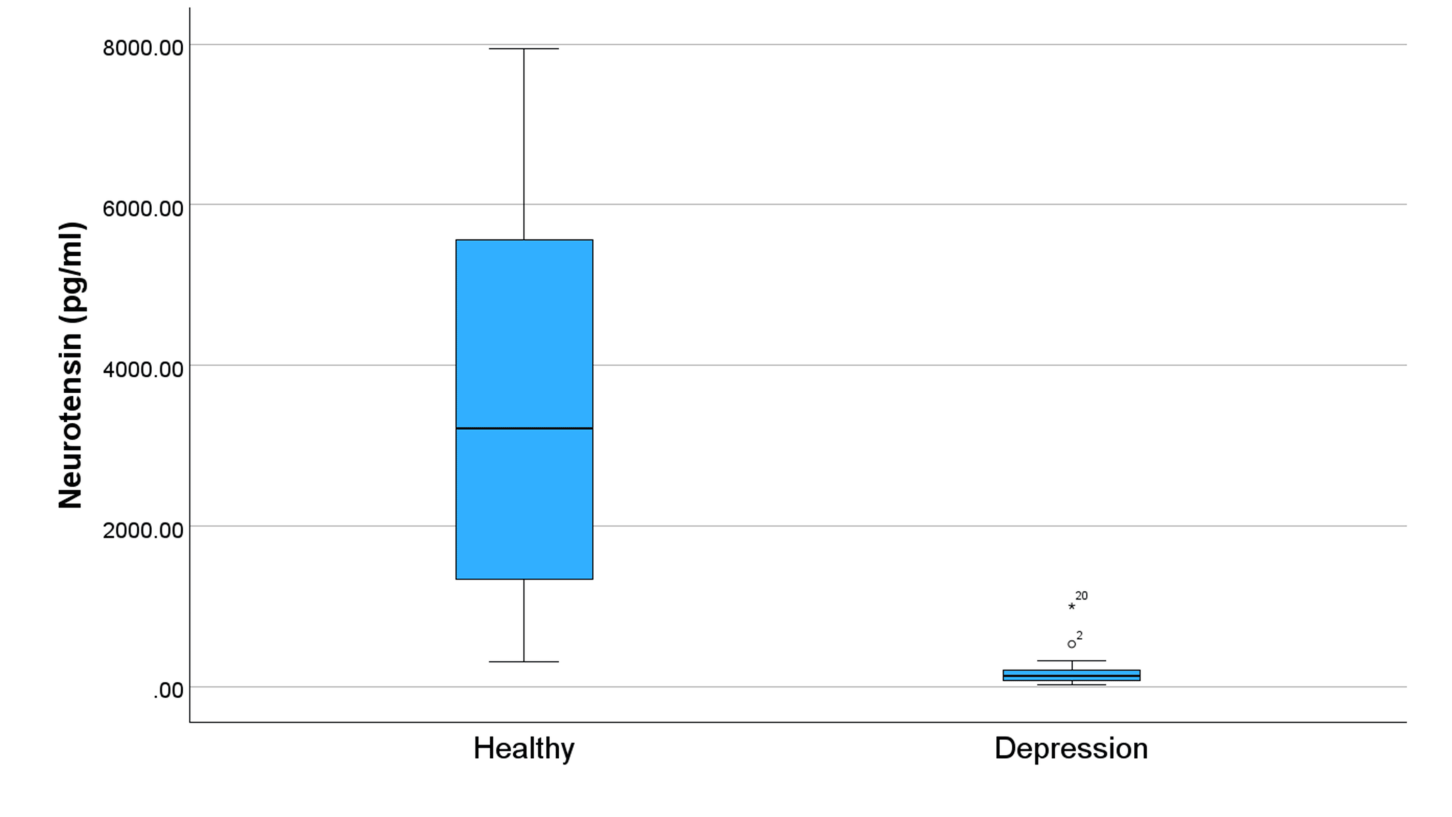
Distribution of neurotensin levels in individuals with depression and healthy controls. The symbols ⚬ and * represent outliers.

**Figure 5:**
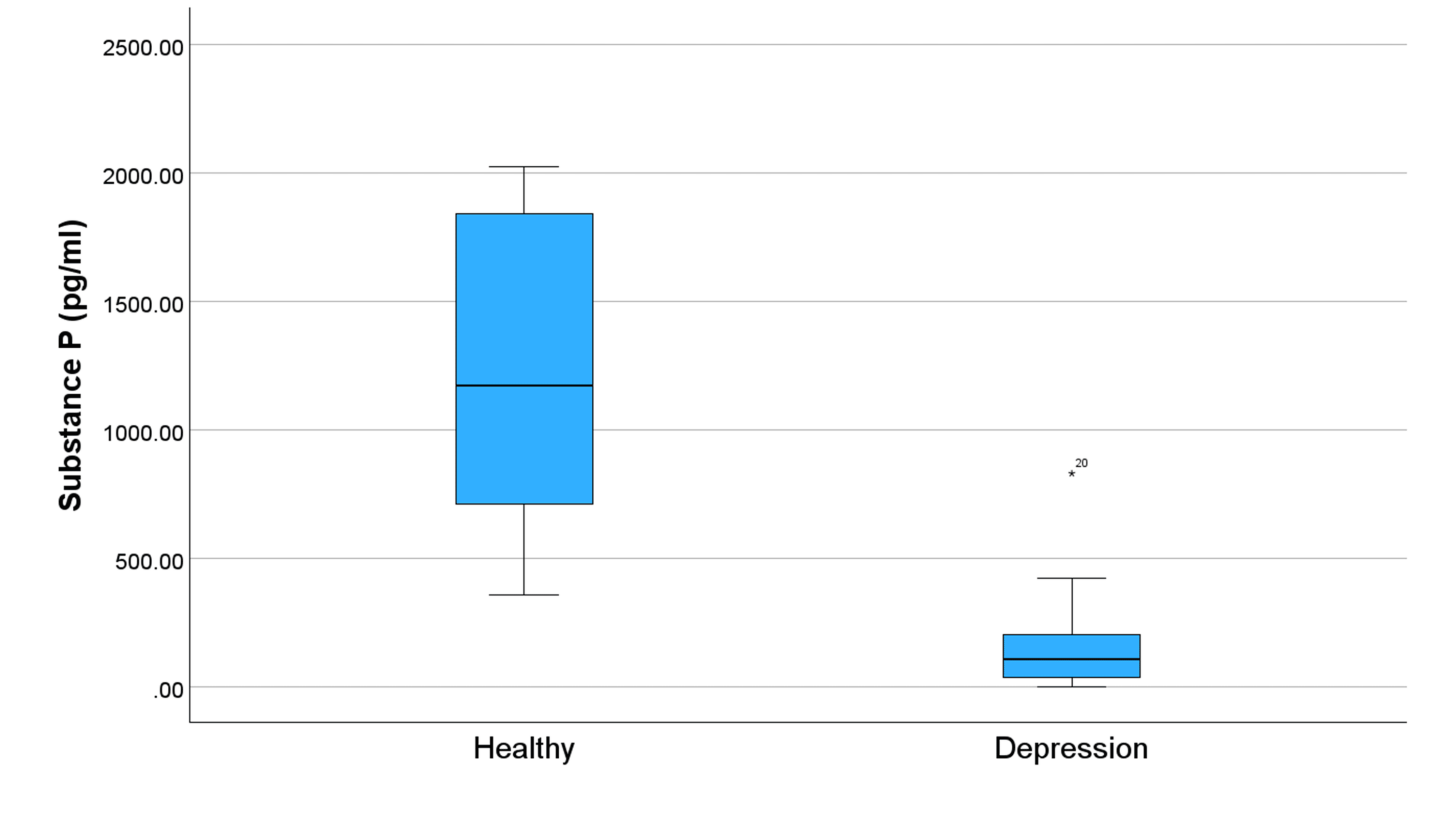
Distribution of substance P levels in individuals with depression and healthy controls. The symbol * represents an outlier.

Figure 1 compares levels of OXT in subjects and controls and shows significantly lower OXT levels in the depression group. This trend of lower levels in the group with depression continues across all studied neuropeptides (Figures 2-5). Specifically, compared to healthy controls, the subjects in Figure 2 exhibit reduced levels of alpha-MSH. Figure 3 details a decrease in beta-endorphin; Figure 4 illustrates lower neurotensin levels; and Figure 5 shows lower substance P levels. Although the significant difference in neuropeptide levels can be seen in all figures, for Figures 2, 3, the deviations in measurements are more noticeable. Collectively, these results suggest that individuals with depression experience a widespread reduction in these biomarkers, which may play a role in the pathophysiology of the disorder.

The statistical analysis confirmed that the differences observed in the neuropeptide levels between the depression and non-depression groups were statistically significant (p < 0.001). The results of the independent t-tests and one-way ANOVA indicate that there are substantial differences in the expression levels of these biomarkers between the depression and control groups. These findings suggest that these neuropeptides could potentially serve as biomarkers for depression.

## Discussion

Neuropeptides have been demonstrated to exert both direct and indirect influences on the pathophysiology of behavioral disorders, including depression, and are therefore being studied as a promising diagnostic tool [20]. This research shows salivary neuropeptide measurements in subjects with depression and healthy controls. The results indicate a significant decrease in levels of all five examined neuropeptides. However, this is highly dependent on the method of measurement used and is in dire need of standardization. Classic examination methods report values in "tens," while our results recorded using the Luminex MAGPIX® System are in the "hundreds," indicating that a more sensitive diagnostic method is necessary to accurately measure salivary neuropeptides.

A recent study by Hidese et al., using the same diagnostic system, focused on plasmatic levels of studied neuropeptides and yielded no significant results across all studied groups [21]. This suggests that measurement and detection sensitivity may significantly influence the observed associations between neuropeptides and psychiatric conditions. Additionally, another study that did not focus on anxiety or depression but measured these neuropeptides in acute stress scenarios, such as in fire recruits, concluded that all neuropeptides increased synchronously during stress events [22].

We recorded a 7.3-fold mean decrease in salivary OXT levels in subjects with depression, aligning with findings focused on plasmatic levels of OXT and depression [23,24]. OXT's role in social bonding and stress regulation may explain its reduced levels in depressive disorders, and its consistent decrease suggests potential clinical applications in developing diagnostic tools or therapeutic interventions that target OXT pathways. Similarly, alpha-MSH has shown associations with affective disorders in animal models, with our study noting a significant deviation in values yet presenting a 3.9-fold decrease in depressive subjects [25]. The deviation in recorded values was significant, and the final mean decrease was the second lowest, preceded only by beta-endorphin, which showed a 2.9-fold reduction. This reduction in alpha-MSH levels might inform future strategies for early detection and personalized treatment approaches in depression.

Older studies have pointed to beta-endorphin as a potential biomarker for a positive treatment response in depression, but our findings indicate significant variability and uncertainty in measured values, suggesting the need for further investigation into its diagnostic utility [26,27]. Despite this variability, the reduction in beta-endorphin could still hold promise for refining therapeutic strategies that enhance endogenous beta-endorphin activity.

Notably, neurotensin levels produced the most considerable decrease among all studied neuropeptides, with a 15.1-fold reduction. A recent study noted that only women showed a positive correlation between neurotensin and perceived stress, anxiety, and depression, aligning with similar findings in animal models [28,29]. The substantial decrease in neurotensin suggests it could serve as a valuable biomarker for developing gender-specific diagnostic tools or treatments for mood disorders.

Yu et al. studied multiple plasma neuropeptides in patients experiencing their first episode of schizophrenia, bipolar disorder, or major depressive disorder, reporting that high neurotensin levels were associated with better executive function, while lower beta-endorphin levels correlated with morning weakness, and reduced OXT levels were linked to more severe psychotic symptoms [6]. All examined neuropeptides, including alpha-MSH, were decreased across these patient groups compared to controls, reinforcing the potential role of neuropeptides in psychiatric conditions.

Substance P, known for its role in pain and stress responses, showed a 6.9-fold decrease in salivary levels in depressive subjects, correlating with published measurements of plasma levels [30]. This substantial reduction highlights its potential as a future diagnostic target for depression. The consistent findings across various studies underscore the need for more precise and sensitive diagnostic methods to accurately assess neuropeptide levels in both saliva and plasma and their implications for psychiatric disorders.

Our study has several limitations. The sample size and participant diversity were limited, restricting the results' reliability and generalizability. The findings are also heavily dependent on the sensitivity and specificity of the diagnostic tools used. Some neuropeptides exhibited high variability in recorded values, potentially affecting the consistency of the results. Additionally, the analysis did not account for various potential confounding factors, such as medication use, diet, physical activity, personal history, or recent life events. The saliva samples were collected only once within a broad time frame, which could introduce variability and limit the accuracy of the biomarker measurements. Furthermore, the cross-sectional design of the study limits our ability to infer causal relationships between neuropeptide levels and depression. Longitudinal studies would be necessary to establish causality.

## Conclusions

In summary, all analyzed neuropeptides (OXT, alpha-MSH, beta-endorphin, neurotensin, and substance P) exhibited significantly lower levels compared to the control group. Based on the obtained results, where the difference in mean concentrations in subjects and controls is p<0.001, it can be emphasized that selected neuropeptides seem to be promising diagnostic biomarkers in the diagnostic process of depression or possibly response to therapy. Nevertheless, further studies are necessary to verify their accuracy and sensitivity, primarily by standardizing the test results, as they are highly variable depending on the method used.
